# Statewide Transfer Coordination and Patient Transfer Rates Among Hospitals During Occupancy Stress

**DOI:** 10.1001/jamanetworkopen.2025.46002

**Published:** 2025-12-01

**Authors:** Mary E. Richert, Guoqing Diao, Alex Mancera, Brittany Badesch, Maniraj Neupane, Roxana Amirahmadi, Sadia H. Sarzynski, Sarah Warner, Joel S. Weissman, Eric Goralnick, Brian J. Franklin, Bruce J. Swihart, Lisa Villarroel, John L. Hick, Steven H. Mitchell, Parneet Kaur, Benjamin Fisher, Sameer S. Kadri

**Affiliations:** 1 Critical Care Medicine Department, National Institutes of Health Clinical Center, Bethesda, Maryland; 2 Critical Care Medicine Branch, National Heart, Lung, and Blood Institute, Bethesda, Maryland; 3 Department of Biostatistics and Bioinformatics, George Washington University, Washington, DC; 4 Grand Island Regional Medical Center, Bryan Health, Grand Island, Nebraska; 5 Center for Surgery and Public Health, Brigham and Women’s Hospital, Harvard Medical School, Boston, Massachusetts; 6 Department of Emergency Medicine, Stanford University, Palo Alto, California; 7 Arizona Department of Health Services, Phoenix; 8 Hennepin Healthcare, University of Minnesota, Minneapolis; 9 Department of Emergency Medicine, University of Washington, Seattle; 10 Data Coordinating Center, University of Utah, Salt Lake City

## Abstract

**Importance:**

Some US states established state medical operations coordination centers (SMOCCs) during the pandemic to coordinate transfers and maximize care delivery when available beds were limited. Understanding their associations might inform their continued value in helping reduce delays and minimize strain during public health emergency conditions.

**Objective:**

To examine the association of SMOCC establishment with adult interhospital transfers (IHTs) during pandemic surges.

**Design, Setting, and Participants:**

This interrupted time series cohort study evaluated hospitalized adults (aged ≥18 years) from 8 US states. Data analysis was completed in March 2025.

**Exposure:**

SMOCC establishment. SMOCC initiation was adjudicated through a published survey and inquiries with state health departments.

**Main Outcomes and Measures:**

Outcome measures were immediate and long-term change in IHT by emergency medical services (EMS) agencies that continuously reported in the National EMS Information Systems database between June 1, 2020, and December 30, 2022. The inflection point (SMOCC establishment) was centered using relative dates while controlling for seasonality. Hospital occupancy stress was measured using daily hospital census and staffed bed counts and weighted by fixed bed capacity. Effect modification by increasing occupancy stress across study states was tested using an interaction term. Findings were validated in several subset analyses.

**Results:**

Across the study’s 8 states (Alaska, Colorado, Idaho, Maryland, North Carolina, Oregon, Utah, and Virginia), 441 709 transfers (median [IQR] age, 61.0 [44.0-73.0] years; 227 982 [51.6%] male) were analyzed, with 321 078 (72.8%) occurring after SMOCC establishment. SMOCC establishment was associated with an immediate increase (rate ratio [RR], 1.35; 95% CI, 1.05-1.74; *P* = .02) followed by a long-term decrease (RR, 0.94; 95% CI, 0.90-0.97; *P* < .001) in transfer rates. A significant increase in transfers per decile increase in occupancy stress was observed 40 weeks into SMOCC establishment (RR, 1.23; 95% CI, 1.06-1.42; *P* = .007). Findings were similar across transfers grouped by urbanicity, mode of transport, patient age, and acuity.

**Conclusions and Relevance:**

In this cohort study of 8 US states, pandemic initiation of a SMOCC was associated with an immediate increase in transfer rates between hospitals of approximately 35% after establishment and, after a potential lag, appeared to meet an increasing demand for transferring patients during surges. These results suggest that activating SMOCCs during large-scale public health emergencies might improve access to care and mitigate transfer gridlocks, but their utility during routine times warrants study.

## Key Points

**Question:**

Did implementation of state medical operations coordination centers (SMOCCs) improve rates of patient transfers among hospitals during pandemic surges?

**Findings:**

In this cohort study of 441 709 interhospital transfers using interrupted time series analysis spanning 8 US states, initiation of a SMOCC during a pandemic was associated with an estimated immediate 35% increase in interhospital transfer rates, and after a lag period, transfers appeared to increase with surging occupancy stress. These findings were validated in transfers that involved rural areas and highest-acuity patients.

**Meaning:**

This study highlights the potential value of SMOCCs in facilitating movement of patients in need of transfer and avoiding gridlocks during large-scale public health emergencies.

## Teaser

This cohort study examines whether the establishment of state medical operations coordination centers was associated with an increase in interhospital patient transfers during pandemic surges.

## Introduction

During the COVID-19 pandemic, many US hospitals experienced capacity strain that impaired patient survival.^1^ One in 4 COVID-19 deaths were attributable to hospital capacity strain.^2^ Capacity strain varied even among hospitals in the same state^3^ and remained detrimental to outcomes^4^ regardless of hospital size or capabilities.^5^ This finding underscores the importance of distributing patients across hospitals (load-balancing) during large-scale emergencies to avoid gridlock and facilitate care.^6,7^ Although interhospital transfers (IHTs) were generally safe during the pandemic,^8^ the process was difficult to operationalize. Bed and staff shortages overwhelmed transfer mechanisms, leading to failed bed requests.^9,10^ As such, IHTs paradoxically decreased during surges,^11^ limiting access to higher-level care. The gridlock was particularly apparent in rural areas^11^ with more limited resources, referral destinations, and transfer capacity, exacerbating the existing US urban-rural health care access gap.^6,7,12^

To improve access to care and unburden overcrowded hospitals during the pandemic, transfer coordination centers called medical operation coordination centers (MOCCs) were implemented at local, regional, and state levels. A recommended framework for establishing MOCCs was released by the US Administration for Preparedness and Response.^13^ State-level MOCCs (SMOCCs) were intended to aggregate patient capacity data and coordinate patient placement for hospitals statewide during times of surge.^13,14^ However, only 19 US states reported establishing a SMOCC.^14^ Despite reports on the successful performance of individual SMOCCs, heterogeneity in operating procedures and transfer policies limits generalizability.^15,16,17^ Postpandemic funding cuts threaten SMOCC operations and prompt discussions regarding their ongoing utility. There is a need for contingency planning for demand-supply imbalances in health care resources.^6,13,18^ Ongoing high occupancy^19^ and staff shortages^20^ threaten transfer capacity during future emergencies.^19^ We conducted an interrupted time series analysis to examine the association between the establishment of SMOCCs and moving patients among hospitals during pandemic surges as a natural experiment to inform their potential role in public health emergencies. We hypothesized that SMOCC establishment would result in higher transfer rates during these high case-load scenarios after implementation.

## Methods

We conducted a retrospective cohort study to evaluate the association of the establishment of SMOCCs with IHT rates overall and during periods of high hospital occupancy. The study was deemed exempt from institutional review board approval by the Office of Human Subjects Research Protections of the National Institutes of Health based on use of exclusively deidentified data; therefore, no informed consent was required. Reporting followed the Strengthening the Reporting of Observational Studies in Epidemiology (STROBE) reporting guideline.^21^ Final data were analyzed March 2025.

### Data Sources

#### SMOCC Data

The status, characteristics, and establishment dates of SMOCCs for eligible patient transfers were determined by existing responses from a published survey of state hospital associations and SMOCC operators.^14^ SMOCCs were defined as (1) centrally aggregating real-time or regularly updated bed capacity data across multiple independent hospitals and/or health systems in a region and (2) managing or coordinating IHTs among independent facilities. Missing data and discrepancies were resolved through follow-up correspondence with state hospital associations or SMOCC operators.

#### IHT Data

IHT data were obtained from the National Emergency Medical Services Information System (NEMSIS) dataset, version 3.4. This database is composed of standardized data on EMS activations from 48 US states, including data from fire department, governmental, hospital, private nonhospital, and tribal services, and leveraged previously for research.^8,22^ Data access was enabled through a preexisting data use agreement between NEMSIS and the National Institutes of Health. In keeping with data use policies, states were kept deidentified by NEMSIS by assigning a dichotomous SMOCC status to transfers across states. Hospital occupancy data were obtained from the US Department of Health and Human Services (DHHS) upon request. The DHHS dataset used was an unredacted precursor to the publicly available dataset COVID-19 Reported Patient Impact and Hospital Capacity by Facility and includes hospital self-reported total inpatient hospital beds, mean total hospital bed use per week, and recorded hospital type.^23^ Access to unsuppressed, but masked, data cells smaller than 5 allowed more accurate estimation of weekly occupancy at very low-volume hospitals. IHT data included patient- and transfer-level characteristics as described below.

### Study Population

IHT for adults (aged ≥18 years) was the primary measured variable. Inpatient IHTs between January 1, 2019, and December 31, 2022, were identified based on predefined criteria (additional details in eTables 1-4 in Supplement 1). The analysis was limited to transfers from states whose SMOCCs were established during the pandemic on or after June 1, 2020, because hospital capacity data were recorded only from that date onward.

#### Patient and Transfer Characteristics

Patient-level characteristics included patient demographics and initial clinical acuity. Transfer-level characteristics included type of transport (ground or air), urbanicity (urban or rural), and transport level of care (basic life support, advanced life support, or specialty critical care) and season.^22^

#### Hospital Occupancy Stress

To quantify the burden of hospital case loads and aggregate at the population level, we defined a new assessment tool (Hospital Occupancy Stress). This variable was created to assess the complex association between changing occupancy and inpatient IHT rates before and after SMOCC initiation. First, we considered hospital occupancy in a given week as high if it met 1 of 2 criteria: (1) occupancy exceeding greater than 80% or (2) occupancy in the top decile for that hospital’s occupancy during the study period and in the top quartile across all hospitals of the same type (eg, short-term acute care) in that week. The 80% cut-off in the first criterion was based on prior models,^24^ and the second criterion captured hospitals with low baseline capacity. To aggregate occupancy across a state in a given week, we calculated the weighted proportion of high occupancy hospitals in each state-week, which was termed *occupancy stress,* and weights were based on hospitals’ fixed bed capacity. Occupancy stress was merged into the dataset by NEMSIS personnel and reported as deciles to keep state identities masked. Given that study states would likely be experiencing different patient volumes at different times, occupancy stress was reported each week as a mean across study states. See eMethods and eFigure 1 in Supplement 1 for further information.

### Statistical Analysis

An interrupted time series analyses was conducted using the negative binomial regression model to assess for the immediate (step) and long-term (trend) changes in the rate of IHTs after SMOCC establishment. The inflection point was date of SMOCC establishment. In accordance with the confidentiality agreement with NEMSIS, which required the concealment of individual state identities, the analysis used only the relative timing from each state’s SMOCC initiation date rather than absolute calendar dates. The unit of the relative time in all analyses is a week. We selected the period so that the IHT data were available for all 8 SMOCC states under consideration. The IHT rates within each week relative to SMOCC establishment represented the outcome.

A 4-level categorical variable was included in the models to account for seasonality. Demographic and transfer-specific variables could not be added as secondary covariates because they characterize the outcome (transfer) and not the population at risk (hospitalized patients). Overall findings were reported at mean level of hospital occupancy stress. Based on prior observations,^11^ we hypothesized that the long-term association of SMOCC establishment with transfers at any given point in time is likely contingent on the degree of occupancy stress encountered by hospitals at that time. To account for scenarios involving widespread hospital caseload surges wherein transfer coordination might be necessary yet operationally challenging, we also examined the association of increasing occupancy stress with the transfer coordinating capability of a newly activated SMOCC. This analysis was performed by testing for effect modification using the product of occupancy stress decile and weeks from SMOCC establishment, which was introduced as an interaction term. The maximum likelihood method was used to estimate the unknown parameters, and the Newey-West method^25^ was used to estimate the SEs to account for potential autocorrelation within time series data. We considered 4 negative binomial regression models, and the model with the smallest Akaike Information Criterion was selected.^26^ Model diagnostics were conducted to evaluate the goodness of fit of the model. The interrupted time series was repeated for subgroups defined by variables such as urbanicity, age, transport mode, level of care, and initial patient acuity. Sensitivity analysis was performed with an unweighted hospital occupancy stress variable (eMethods in Supplement 1). The threshold for statistical significance was *P* < .05. All analyses were conducted using R software, version 4.4.2 (R Foundation for Statistical Computing) (eMethods in Supplement 1).

## Results

### Demographics of IHTs

Across the 8 states, 441 709 total IHTs (median [IQR] age, 61.0 [44.0-73.0] years; 227 982 [51.6%] male and 213 727 [48.4%] female) were reported in NEMSIS between June 1, 2020, and December 31, 2022. Distributions of transfer characteristics are reported in Table 1. Of the transfers, 73 089 (16.5%) were considered emergent and 28 392 (6.4%) of critical acuity based on EMS impressions. Overall, the median (IQR) transport time was 55 (39.0-82.6) minutes. A total of 319 957 transfers (72.4%) originated from urban area hospitals, 395 996 (89.7%) were by ground transport, 278 552 (63.1%) using advanced life support, and 105 249 (23.8%) using specialty critical care ambulances. A total of 120 631 transfers (27.3%) occurred before MOCC establishment, and the remaining 321 078 (72.7%) occurred after MOCC establishment. Patient demographics and transport characteristics were similar between transfers before and after SMOCC establishment (Table 1) and across included and excluded states (eTable 5 in Supplement 1).

**Table 1.** Baseline Characteristics of Interhospital Transfers Originating in 8 Study States

Characteristic	No. (%) of encounters		
	Overall (N = 441 709)	Before SMOCC establishment (n = 120 631)	After SMOCC establishment (n = 321 078)
Age, median (IQR), y	61.0 (44.0-73.0)	61.0 (43.0-73.0)	61.0 (44.0-74.0)
Age group, y			
18-44	114 089 (25.8)	32 102 (26.6)	81 987 (25.5)
45-61	109 884 (24.9)	30 323 (25.1)	79 561 (24.8)
62-73	108 398 (24.5)	29 599 (24.5)	78 799 (24.5)
≥74	119 011 (26.9)	31 289 (25.9)	87 722 (27.3)
Sex			
Female	209 353 (47.4)	56 494 (46.8)	152 859 (47.6)
Male	227 982 (51.6)	62 678 (52.0)	165 304 (51.5)
Unreported	4374 (1.0)	1459 (1.2)	2915 (0.9)
Urbanicity			
Rural	114 307 (25.9)	30 056 (24.9)	84 251 (26.2)
Urban	319 957 (72.4)	88 938 (73.7)	231 019 (72.0)
Unreported	7445 (1.7)	1637 (1.4)	5808 (1.8)
US Census region			
South	190 072 (43.0)	39 922 (33.1)	150 150 (46.8)
West	251 637 (57.0)	80 709 (66.9)	170 928 (53.2)
Initial patient acuity			
Lower (green)	195 286 (44.2)	49 160 (40.8)	146 126 (45.5)
Emergent (yellow)	73 089 (16.5)	20 536 (17.0)	52 553 (16.4)
Critical (red)	28 392 (6.4)	7175 (5.9)	21 217 (6.6)
Dead on arrival (black)	98 (0.0)	25 (0.0)	73 (0.0)
Unreported	144 844 (32.8)	43 735 (36.3)	101 109 (31.5)
Transport mode			
Air	45 713 (10.3)	10 407 (8.6)	35 306 (11.0)
Ground	395 996 (89.7)	110 224 (91.4)	285 772 (89.0)
Transport level of care			
Advanced life support	278 552 (63.1)	86 549 (71.7)	192 003 (59.8)
Basic life support	57 908 (13.1)	11 991 (9.9)	45 917 (14.3)
Specialty critical care	105 249 (23.8)	22 091 (18.3)	83 158 (25.9)
Season			
Fall	128 018 (29.0)	27 373 (22.7)	100 645 (31.3)
Spring	77 261 (17.5)	13 297 (11.0)	63 964 (19.9)
Summer	142 723 (32.3)	66 235 (54.9)	76 488 (23.8)
Winter	93 707 (21.2)	13 726 (11.4)	79 981 (24.9)
Transport time, min			
Median (IQR)	55.0 (39.0-82.6)	54.9 (38.7-82.8)	55.0 (39.2-82.5)
Unreported	1564 (0.4)	428 (0.4)	1136 (0.4)

Abbreviation: SMOCC, state medical operations coordination center.

### SMOCC Characteristics

**Figure 1.**
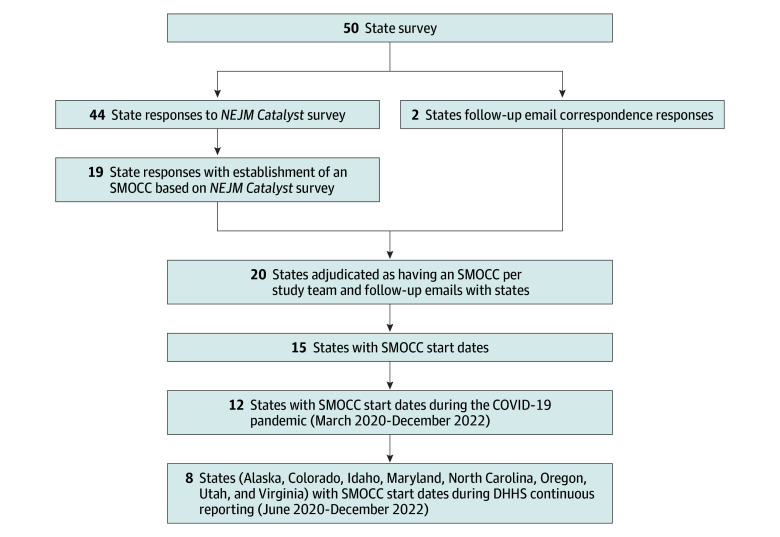
Process of Selecting Study States With State-Level Medical Operations Coordination Centers (SMOCCs) This flowchart shows the steps involved in SMOCC study state selection. *NEJM Catalyst* survey refers to the study by Franklin et al.^14^ The US Department of Health and Human Services (DHHS) occupancy data from the COVID-19 Reported Patient Impact and Hospital Capacity by Facility dataset.

Of the 44 states that responded to the published survey, 19 reported having established a SMOCC during the pandemic and 1 additional state was adjudicated as such after correspondence with state hospital associations. Eight of these states (Alaska, Colorado, Idaho, Maryland, North Carolina, Utah, and Virginia) reported establishing a SMOCC during the pandemic after reporting of occupancy data and comprised our cohort of study states (Figure 1). The need for consistent data on occupancy both before and after SMOCC establishment led to exclusion of states such as Washington, Arizona, and Minnesota, with previous reports of early adoption and successful operation of SMOCCs.^6,15,16,17^

The design features of the SMOCCs varied across the 8 study states (Table 2). All 8 study states were within the Southern or Western US. Operational characteristics included encompassing the entire state (n = 7) vs a subregion of the state (Virginia). Six SMOCCs were active only during times of patient surges, whereas 2 states were continuously operating. Funding for the SMOCCs varied among federal, state, and other emergency preparedness funding mechanisms. The organizations responsible for daily operations included state health departments (n = 2) and state hospital associations (n = 2). The remaining 4 states reported a combination of state emergency management infrastructure and/or where state or regional health care systems assumed responsibility of SMOCC operations. Participation in the SMOCC was voluntary for all hospitals in all states. Additionally, no SMOCC mandated sites to accept patients. All 8 SMOCCs transferred patients with and without COVID-19. Five SMOCCs disregarded patient insurance status, 2 disregarded geography, and 1 disregarded patient or surrogate preferences in placing patients. All but 1 SMOCC had the ability to consult a physician, and all but 1 manually collected data. As of the time of survey reporting (June 2022 to April 2023), only 2 of 8 states still had operating SMOCCs.

**Table 2.** SMOCC Characteristics of Study Sites^a^

Characteristics	No. of states
**Operations**	
SMOCC region	
Entire state	7
Part of state	1
Geographic region	
South	3
West	5
Funding (>1 selection per state)	
Federal	5
State	2
Other	3
Entity responsible for daily operations	
State health department	2
State hospital association	2
Other	4
Activation criteria	
Surge	6
Continuous	2
Operation status as of April 2023	
Currently operating	2
Previously operating	6
**Design variables**	
Ability to consult physician	
Never	1
Sometimes or always	7
Bed and/or resource availability data	
Manual recurrent data submission	7
Real-time, automatically updated data	1
Mechanism to prioritize patient transfers when requests exceeded available beds	
Yes	5
No	3
**Transfer coordination framework**	
Hospital participation mandatory	
Mandatory	0
Voluntary	8
Hospital participation within area	
Some hospitals	2
All hospitals	6
Ability to mandate site to accept patient	
No	8
Yes	0
Types of patients	
COVID-19 only	0
COVID-19 and non–COVID-19	8
Factors in patient placement	
Disregards geography	2
Disregards insurance status	5
Disregards patient and/or family preference	1

Abbreviation: SMOCC, state medical operations coordination center.

^a^ Results adapted from Franklin et al^14^ National Survey Response Data.

### Association of SMOCC Establishment With Transfers and Interaction by Occupancy Stress

The association between SMOCC establishment and transfers at mean occupancy stress are detailed in Table 3 and eTable 6 in Supplement 1, which presents the results under the model selected by Akaike information criterion. Before establishment of SMOCCs, occupancy stress showed no association with transfer rates (relative risk [RR], 0.99; 95% CI, 0.94-1.04; *P* = .68). Because the model after establishment of SMOCCs included interactions among occupancy stress, the establishment of SMOCCs, and time since intervention, the immediate and long-term associations of SMOCCs on transfers depend on occupancy stress level. Immediately on SMOCC establishment, there was an increase in the numbers of transfers (RR, 1.35; 95% CI, 1.05-1.74; *P* = .02). In other words, at mean occupancy stress, the SMOCC implementation resulted in a median (IQR) 35% (5%-74%) increase in transfer rates after a SMOCC was established. Notably this upswing was followed by a negative long-term (RR, 0.94; 95% CI, 0.90-0.97; *P* < .001) prolonged downtrend in transfers at mean occupancy stress. Whereas the interaction of long-term post-SMOCC and hospital occupancy stress (RR, 1.00; 95% CI, 0.99-1.01, *P* = .36) implies the long-term association increases by 0.4% with each unit increase in stress decile, this finding was not statistically significant (eTable 6 in Supplement 1).

**Table 3.** Immediate and Long-Term Changes on Transfer Rates After SMOCC Initiation at Mean Occupancy Stress for Primary and Subgroup Analyses^a^

Variable	Immediate change		Long-term change	
	Rate ratio (95% CI)	*P* value	Rate ratio (95% CI)	*P* value
Primary cohort (8 states)	1.35 (1.05-1.74)	.02	0.94 (0.90-0.97)	<.001
Subgroups				
Location				
Rural	1.34 (0.99-1.80)	.06	0.94 (0.91-0.98)	.002
Urban	1.34 (1.07-1.69)	.01	0.93 (0.90-0.97)	<.001
Mode of transport				
Air	1.30 (0.97-1.75)	.08	0.94 (0.91-0.96)	<.001
Ground	1.36 (1.05-1.76)	.02	0.94 (0.90-0.97)	<.001
Transport level of care^b^				
Advanced life support	1.25 (0.99-1.57)	.056	0.95 (0.92-0.98)	<.001
Basic life support	1.26 (1.03-1.55)	.02	0.95 (0.91-0.98)	.004
Specialty critical care	1.52 (1.07-2.15)	.02	0.92 (0.88-0.96)	<.001
Initial patient acuity				
Lower acuity (green)	1.46 (1.15-1.86)	.002	0.92 (0.88-0.96)	<.001
Emergency (yellow)	1.18 (0.96-1.46)	.11	0.95 (0.92-0.97)	<.001
Critical (red)	1.14 (0.88-1.44)	.29	0.96 (0.93-0.98)	<.001
Age group, y				
18-44	1.28 (0.96-1.71)	.10	0.95 (0.92-0.98)	.004
45-61	1.35 (1.03-1.76)	.03	0.93 (0.90-0.97)	<.001
62-73	1.42 (1.06-1.89)	.02	0.93 (0.90-0.96)	<.001
≥74	1.41 (1.15-1.73)	.001	0.94 (0.90-0.97)	<.001

Abbreviation: SMOCC, state medical operations coordination center.

^a^ Model estimates are obtained at mean occupancy stress.

^b^ Transport level of care denoting dead on arrival was not included in this model.

**Figure 2.**
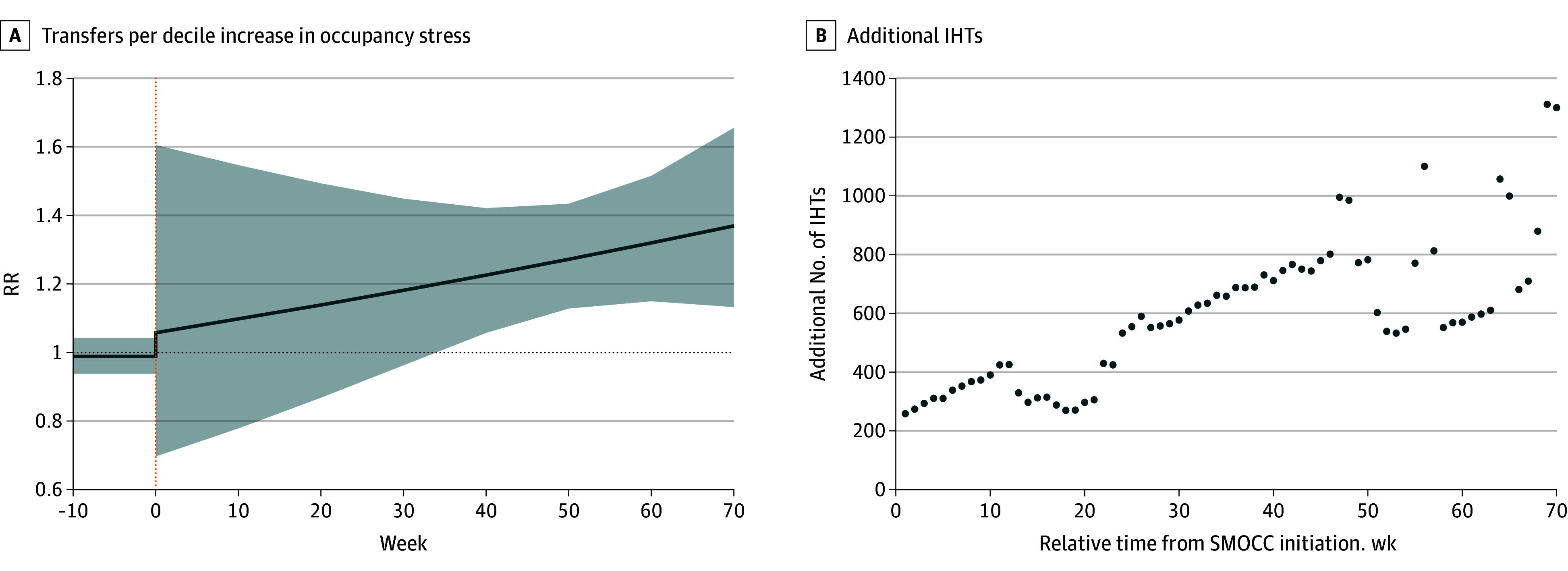
Change in Interhospital Transfers (IHT) With Increasing Hospital Occupancy Stress Based on Time Since State-Level Medical Operations Coordination Center (SMOCC) Establishment A, The log rate ratios (RRs) are the (time-dependent) effect of occupancy stress on the IHTs. Before SMOCC establishment, no association was observed in transfers per decile increase in occupancy stress (RR, 0.99; 95% CI, 0.94-1.04; *P* = .68). However, by 40 weeks the lower bound of the CI stays above 1 (RR, 1.23; 95% CI, 1.06-1.42; *P* = .007). Shaded areas are 95% CIs; dotted orange line, SMOCC initiation. B, Additional numbers of IHT would have occurred had the corresponding occupancy stress level been increased by 1 decile over time from week 0 to week 70 since the SMOCC initiation.

The association between transfers and occupancy stress over time can also be seen in Figure 2 and eTable 7 and eFigures 2 and 3 in Supplement 1). Again, before the establishment of SMOCCs, occupancy stress was not associated with transfer rates (RR, 0.99; 95% CI, 0.94-1.04; *P* = .68) (Figure 2A; eFigure 3 in Supplement 1). Observing this association after SMOCC establishment, we found that transfers increased over time, with increasing occupancy stress levels that became significant by 40 weeks (RR, 1.23; 95% CI, 1.06-1.42; *P* = .007) (Figure 2A; eFigure 3 and eTable 7 in Supplement 1). Additionally, we graphically represented the increase in transfers per decile increase in occupancy stress for weeks 1 to 70 after the establishment of MOCCs in Figure 2B, corresponding to a median (IQR) of 596 (377-741) additional transfers. Sensitivity analysis performed with unweighted hospital occupancy stress variable also demonstrated similar results (eTables 8 and 9 in Supplement 1).

### Subgroup Analysis

At mean occupancy stress, all subgroups showed increases in transfers immediately after SMOCC establishment, which were statistically significant for urban, ground transport, basic life support, specialty clinical care transfers, older age cohorts, and the lowest acuity transfer subsets (Table 3). With increasing occupancy stress, most subgroups demonstrated increases in transfer, which became statistically significant (lower bound of 95% CIs above 1 in eFigure 3 in Supplement 1) by 30 to 50 weeks after the establishment of SMOCCs.

## Discussion

This interrupted time series analysis highlights the value of SMOCCs during the COVID-19 pandemic and provides lessons for future health care demand-supply imbalances. In a cohort of 8 Southern and Western states, the rate of weekly IHTs increased approximately 35% immediately after SMOCC establishment at mean occupancy. Although it is difficult to attribute the increase to a specific SMOCC policy, it may represent a fulfillment of a previously unmet backlog of transfers. Notably, at mean occupancy, the short-term increase was followed by a decrease in transfers. However, hypothesizing a more vital role for SMOCCs during surges, we also examined whether SMOCCS modified the association between occupancy stress and transfers. Prior to SMOCC establishment, we observed no association between occupancy stress and transfers. After SMOCC implementation, transfers increased with increasing occupancy stress, becoming significant by approximately 40 weeks after implementation. This association was observed across most subgroups (Figure 2A; eFigure 3 in Supplement 1) and corresponded to a mean of 596 additional transfers per decile increase in occupancy stress (Figure 2B). Although the 40-week threshold arises from empiric observation, a plausible hypothesis is that this lag reflects the time for SMOCCs to improve communication and operational efficiency. Further work is needed to validate this finding and explore factors influencing this implementation period.

These findings from the pandemic align with prior reports^6,16,17,27^ on individual SMOCC performance and provide insights into future events. The pandemic may have ended, but hospital occupancy continues to exceed prepandemic baselines^19^ and staffing numbers have not recovered.^20^ This fosters conditions in which hospitals may struggle to handle fluctuations in demand, especially during sudden and overwhelming stress. Our study found that, before SMOCC establishment, states were not able to increase transfer in response to increasing hospital occupancy. We hypothesize that similar limitations may have existed in states that never implemented a SMOCC. A previous study^11^ similarly showed that transfers did not proportionally increase during pandemic strain conditions in area hospitals. Surge capacity expansions for potential combat operations (such as those assessed under the National Disaster Medical System Pilot)^28^ or disasters (fires,^29^ hurricanes,^30,31^ viral surges,^32^ or terrorism^33,34^) are contingent on functioning load-balancing processes. However, deficiencies within existing transfer systems were exposed when encountering a gridlock during pandemic surges^11^ and likely persist. Such events could rapidly exceed transfer capacity and might benefit from centralized communication, data visualization, and troubleshooting.

Our study has important implications for rural areas with large representation within the Southern and Western US. Rural hospitals, particularly unaffiliated ones, have had difficulty transferring patients.^6,7,11,35^ A prior 3-state cohort noted that more than 50% of transfer requests during the pandemic originated from rural hospitals.^6^ Although small urban hospitals were able to flex transfers nearly 50% during pandemic surges, small rural hospitals did not.^11^ Our finding of the eventual reversal of the occupancy stress–transfer association from null to positive after SMOCC implementation extended to rural area transfers (eFigure 3 in Supplement 1) and offers hope. Facilitating transfers for higher level of care from resource-limited rural hospitals may help improve outcomes during strained times.^5^ The long-term downtrend in transfers at routine occupancy (Table 2) raises questions on the utility of SMOCCs in facilitating daily transfers during routine times in the long term. However, the ability to adapt and boost transfers during higher occupancy stress (Figure 2; eFigure 3 in Supplement 1) suggests a potential role for SMOCCS in areas with health care systems under long-term stress. Given that low-resourced settings might particularly benefit from such ongoing assistance, dedicated study of SMOCC performance is warranted.

Our study brings up important funding considerations for nationwide emergency preparedness amid an uncertain future for SMOCCs. During the COVID-19 pandemic, many states had funding provided by a combination of federal (CARES Act, Hospital Preparedness Program, and American Rescue Plan Act) and state emergency funds.^6^ The funding mechanisms for the states in our study varied among federal, state, and other funding sources, consistent with prior reports^14,15,16^ (Table 2). Unfortunately, many were one-time emergency response measures and have been significantly reduced or discontinued. Notably, we found that 6 of the 8 study MOCCs were no longer operational at the time of survey reporting. Nonetheless, the immediate association of SMOCC establishment on transfer rates suggests that activation during emergency events could enable rapid patient redistribution. Such redistribution might be achievable at lower cost with intermittent training, reservist SMOCC staff, and existing health systems and EMS agencies enabling transfers. However, the decrease in transfers after the immediate spike and the postlag reversal in transfer capabilities under stress suggests that coordination systems may require time to become fully operational. Developing cooperative agreements and building an effective coordination system is complex; having infrastructure and situational operating procedures established in advance may allow for more rapid and effective SMOCC-mediated patient redistribution in future crises.

### Strengths and Limitations

Our study has many strengths. To our knowledge, it is the only study to date to evaluate aggregate SMOCC performance during the COVID-19 pandemic. We used survey data to objectively select 8 eligible states with a diverse range of operating characteristics, making our findings more generalizable and less subject to publication bias than prior reports on SMOCC performance. We created and used a novel metric for hospital occupancy stress that can be used beyond the COVID-19 pandemic,^1^ increasing its future applicability for alternative transfer scenarios. The metric was designed to capture relative stress encountered across US hospitals that operate at disparate baseline occupancy levels. Strengths of the analysis include use of relative dates (enabling multistate evaluation of SMOCC performance while controlling for seasonality) and an interaction term to enable a more accurate representation of SMOCC performance during high caseload stress.

Our study also has limitations. Our study does not include SMOCCs in the Northeast and Midwest and may not be nationally representative. However, less than half of all US states reported having deployed a SMOCC, and in our study, patient and transfer characteristics were comparable between included and excluded states.^14^ The analytical need to overlap MOCC establishment dates within the timeframe when DHHS bed occupancy data were available potentially led to selection bias in state inclusion. Our methods excluded some early SMOCC adopters, such as Washington,^16^ Minnesota,^15^ and Arizona,^17,35^ potentially underrepresenting the broader influence of SMOCC implementation nationwide, underestimating effect size, and affecting generalizability. Daily census and SMOCC status were self-reported, which could have introduced bias. Differences in state policy and SMOCC design features across study states preclude attribution to a specific policy or feature. The analysis might not account for some patients transferred among states and those not transported by EMS mechanisms (eg, hospital-based transport), which might introduce bias. However, the NEMSIS dataset captures most hospital-to-hospital transfers and licensed transports in each state based on EMS activations.^8,22^ Patient demographics and other characteristics could not be included as secondary covariates. However, we conducted 14 subset analyses, yielding generally consistent findings. Additionally, due to licensing agreements with NEMSIS, we were required to mask identities of individual states. To prevent disclosure of transfer locations, state-level occupancy data were aggregated into occupancy deciles rather than reported individually. As a result, we were unable to provide continuous estimates for hospital occupancy stress.

## Conclusions

In this cohort study of IHTs from 8 US states during the COVID-19 pandemic, transfer rates increased by 35% after SMOCC initiation at mean occupancy stress, emphasizing the potential value of activating SMOCCs during discrete events that necessitate rapid, large-scale transfer coordination. This study also supports the hypothesis that SMOCCs may help with patient load-balancing during hospital strain, with transfer rates appearing to increase in response to occupancy stress approximately 40 weeks after implementation. These findings provide a strong basis to reappraise the ongoing benefit of SMOCCs as a critical contingency and optimize their configuration and functions to maximize patient benefit. Further research should examine barriers to SMOCC adoption in other states and further elucidate the role of SMOCCs in improving patient flow in low-resource areas of the country.

## Notes

Supplement 1. **eMethods. **Supplementary Methods **eTable 1**. Codes Defining Treating Patient **eTable 2**. Codes Defining Transported Patients **eTable 3. **Codes Defining Interhospital Transported Patients **eTable 4. **Codes Defining Interhospital Transport Mode **eFigure 1. **Graphic of Hospital Occupancy Stress Variable Creation **eTable 5.** Overall Interhospital Transfer Characteristics During Study Period **eTable 6.** Full Interrupted Times Series Analysis of Aggregated Eight SMOCC Study States **eTable 7. **Hospital Occupancy Stress Effect on Interhospital Transfer (IHT) Rate Over Time **eTable 8. **Sensitivity Analysis with Unweighted Hospital Occupancy Stress Effect on Interhospital Transfer (IHT) Rate Over Tim**e** **eTable 9.** Sensitivity Analysis with Unweighted Hospital Occupancy Stress for Full Interrupted Times Series Analysis of Aggregated Eight SMOCC Study States **eFigure 2. **Cumulative Interhospital Transfers and 95% pointwise confidence bands over time since SMOCC initiation by Hospital Occupancy Stress decile level and by season. **eFigure 3. **Subgroup Analysis for Change in Interhospital Transfers with Increasing Occupancy Stress and Interaction by Time Since SMOCC Establishment **eFigure 4. **Plot of deviance residuals against occupancy stress
eReferences

Supplement 2.
Data Sharing Statement
