# Supplementary material for: Statewide Transfer Coordination and Patient Transfer Rates Among Hospitals During Occupancy Stress
Source: JAMA Netw Open. 2025 Dec 1;8(12):e2546002. doi: 10.1001/jamanetworkopen.2025.46002 (PMC12670198; doi:10.1001/jamanetworkopen.2025.46002)
Supplement: Supplement 1. — eMethods. Supplementary Methods eTable 1. Codes Defining Treating Patient eTable 2. Codes Defining Transported Patients eTable 3. Codes Defining Interhospital Transported Patients eTable 4. Codes Defining Interhospital Transport Mode eFigure 1. Graphic of Hospital Occupancy Stress Variable Creation eTable 5. Overall Interhospital Transfer Characteristics During Study Period eTable 6. Full Interrupted Times Series Analysis of Aggregated Eight SMOCC Study States eTable 7. Hospital Occupancy Stress Effect on Interhospital Transfer (IHT) Rate Over Time eTable 8. Sensitivity Analysis with Unweighted Hospital Occupancy Stress Effect on Interhospital Transfer (IHT) Rate Over Time eTable 9. Sensitivity Analysis with Unweighted Hospital Occupancy Stress for Full Interrupted Times Series Analysis of Aggregated Eight SMOCC Study States eFigure 2. Cumulative Interhospital Transfers and 95% pointwise confidence bands over time since SMOCC initiation by Hospital Occupancy Stress decile level and by season. eFigure 3. Subgroup Analysis for Change in Interhospital Transfers with Increasing Occupancy Stress and Interaction by Time Since SMOCC Establishment eFigure 4. Plot of deviance residuals against occupancy stress eReferences [file jamanetwopen-e2546002-s001.pdf]

Supplemental Online Content

Richert ME, Diao G, Mancera A, et al. Statewide transfer coordination and patient transfer rates among hospitals during pandemic surges. *JAMA Netw Open*. 2025;8(12):e2546002. doi:10.1001/jamanetworkopen.2025.46002

eMethods. Supplementary Methods

eTable 1. Codes Defining Treating Patient

eTable 2. Codes Defining Transported Patients

eTable 3. Codes Defining Interhospital Transported Patients

eTable 4. Codes Defining Interhospital Transport Mode

eFigure 1. Graphic of Hospital Occupancy Stress Variable Creation

eTable 5. Overall Interhospital Transfer Characteristics During Study Period

eTable 6. Full Interrupted Times Series Analysis of Aggregated Eight SMOCC Study States

eTable 7. Hospital Occupancy Stress Effect on Interhospital Transfer (IHT) Rate Over Time

eTable 8. Sensitivity Analysis with Unweighted Hospital Occupancy Stress Effect on Interhospital Transfer (IHT) Rate Over Time

eTable 9. Sensitivity Analysis with Unweighted Hospital Occupancy Stress for Full Interrupted Times Series Analysis of Aggregated Eight SMOCC Study States

eFigure 2. Cumulative Interhospital Transfers and 95% pointwise confidence bands over time since SMOCC initiation by Hospital Occupancy Stress decile level and by season.

eFigure 3. Subgroup Analysis for Change in Interhospital Transfers with Increasing Occupancy Stress and Interaction by Time Since SMOCC Establishment

eFigure 4. Plot of deviance residuals against occupancy stress

eReferences

This supplemental material has been provided by the authors to give readers additional information about their work.

© 2025 Richert ME et al. *JAMA Network Open.*

eMethods. Supplementary Methods

Interhospital transfer (IHT) data was obtained from the National Emergency Medical Services Information System (NEMSIS) Dataset version 3.4. This database is composed of standardized data on emergency medical services (EMS) activations from 48 U.S. states, including data from fire department, governmental, hospital, private non-hospital, and tribal services.[1]

Data Curation:

Identifying Interhospital Transfer (IHT) Study Population: Interhospital transfer patients were determined using the NEMSIS eDisposition_12 code 4212033 identifying "Patient Treated, Transported by this EMS Unit". This filtered out patients deceased on arrival, no longer in the facility, or those not treated by EMS. These patients were further selected by NEMSIS eResponse_05 (Type of Service Requested) codes 2205005 "Interfacility Transport" or 2205007 "Medical Transport", eScene_09 (Incident Location Type) codes Y92.23 (Hospital) or Y92.230 (Patient Room in Hospital), eDisposition_21 (Type of Destination) codes 4221003 (Hospital-Emergency Department), 4221005(Hospital-Non-Emergency Department Bed), and eResponse_07 (Primary Role of This Unit) codes 2207003 (Ground Transport), 2207011 (Air Transport-Helicopter), 2207013(Air Transport-Fixed Wing) to filter out transfers not associated with interhospital transport.

eTable 1. Codes Defining Treating Patient

eDisposition_12 code	Incident/Patient Disposition
4212033	Patient Treated, Transported by this EMS Unit

eTable 2. Codes Defining Transported Patients

eResponse_05	Type of Service Requested
2205005	Interfacility Transport
2205007	Medical Transport

eTable 3. Codes Defining Interhospital Transported Patients

eScene_09	Incident Location Type
Y92.23	Hospital as the place of occurrence of the external cause
Y92.230	Patient room in hospital as the place of occurrence of the external cause

eDisposition_21	Type of Destination
4221003	Hospital-Emergency Department
4221005	Hospital-Non-Emergency Department Bed

eTable 4. Codes Defining Interhospital Transport Mode

eResponse_07	Primary Role of the Unit
2207003	Ground Transport
2207011	Air Transport-Helicopter
2207013	Air Transport-Fixed Wing

Hospital Stress Occupancy Variable Creation:

In an effort to create an encompassing metric for occupancy caseload stress, we utilized the U.S Department of Health and Human Services (HHS) dataset Protect Database "COVID-19 Reported Patient Impact and Hospital Capacity by Facility".[2] Only hospitals that reported inpatient bed utilization were used. Missing data was not imputed. We used the average weekly number of staffed adult inpatient beds used and the number of staffed adult inpatient beds available, per state, per hospital type to determine weekly staffed bed occupancy counts.

We first defined a hospital as under "stressed" conditions if the hospital had greater than 80% weekly staffed bed occupancy based on a CDC Morbidity and Mortality Weekly report indicating a hospital as under "strained" condition if identified occupancy is > 80%. However, to create an encompassing metric in consideration of hospitals that typically operate at lower capacity but could still feel strain from increased patient numbers, we considered an alternative criteria that for a specific hospital to be in "occupancy stress" it must meet two criteria: 1) occupancy in a given week was in the top decile of that hospital's occupancy distribution over study period AND 2) occupancy in the top quartile of occupancy distribution for all hospitals of the same type in that week. We then weighted this value by staffed bed number and separated into surge deciles per state, per week.

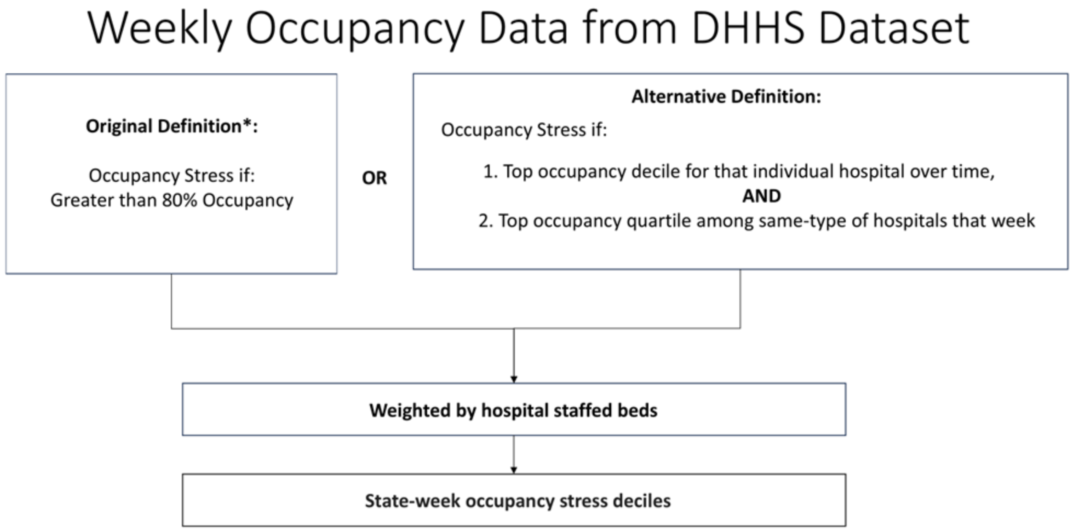

*French et al. MMWR 2021

eFigure 1: Graphic of Hospital Occupancy Stress Variable Creation.

© 2025 Richert ME et al. *JAMA Network Open.*

Statistical Analysis:

We conducted the interrupted time series (ITS) analysis using the negative binomial (NB) regression model to assess the effects of SMOCC initiation, COVID occupancy capacity, and their interaction effects on the number of inter-hospital transfers (IHTs). A four-level categorical variable taking values ‘spring’, ‘summer’, ‘fall’, and ‘winter’, was included in the model to account for seasonality. Due to confidentiality concerns, only the relative time from the SMOCC initiation date for the eight SMOCC states (Colorado, Maryland, Alaska, Utah, Virginia, Idaho, North Carolina, and Oregon) was used rather than the calendar time. The unit of the relative time in all analyses is week. We selected the period such that the IHTs data were available for all eight SMOCC states under consideration.

We denote D_t a dummy variable indicating observations collected before (=0) and after (=1) the SMOCC initiation, P_t a continuous variable indicating the time passed since the SMOCC initiation and taking value 0 before MOCC initiation, X_t the occupancy capacity variable, and $Z_t = (Z_{t1}, Z_{t2}, Z_{t3})$ three dummy variables taking value 1 corresponding to ‘spring’ (March-May), ‘summer’ (June-August), and ‘fall’ (September-November), respectively, and value 0 otherwise. For the seasonality variable, ‘winter’ (December-February) was treated as the reference. Numerical values 1-10 were allocated to the cumulative occupancy variable corresponding to the 10 deciles from “0-9” to “90-100”. Since only the relative dates could be used in the analysis, we calculated the average of the cumulative occupancy across the study states within each week (relative to the SMOCC initiation). For the seasonality variable, we calculate the proportions of each season within the week.

We fit the following four NB regression models using the log-link function

$$\log\mu_t = \beta_0 + \beta_1 t + \beta_2 t^2 + \beta_3 D_t + \beta_4 P_t + \beta_5 X_t + \beta_6 X_t D_t + \gamma^T Z_t, \quad (1)$$

$$\log\mu_t = \beta_0 + \beta_1 t + \beta_2 t^2 + \beta_3 D_t + \beta_4 P_t + \beta_5 X_t + \beta_6 X_t D_t + \beta_7 X_t P_t + \gamma^T Z_t, \quad (2)$$

$$\log\mu_t = \beta_0 + \beta_1 t + \beta_2 t^2 + \beta_3 D_t + \beta_4 P_t + \beta_5 P_t^2 + \beta_6 X_t + \beta_7 X_t D_t + \gamma^T Z_t, \quad (3)$$

and

$$\log\mu_t = \beta_0 + \beta_1 t + \beta_2 t^2 + \beta_3 D_t + \beta_4 P_t + \beta_5 P_t^2 + \beta_6 X_t + \beta_7 X_t D_t + \beta_8 X_t P_t + \beta_9 X_t P_t^2 + \gamma^T Z_t, \quad (4)$$

where μ_t is the mean value of the number of IHTs in week t. Here, models (1) and (2) include the linear term of P_t, whereas models (3) and (4) include both linear and quadratic terms of P_t. On the other hand, models (1) and (3) include the interaction between X_t and D_t only, whereas models (2) and (4) include the interaction between X_t and and the interaction between X_t and (D_t, P_t, P_t^2), respectively. Under model (4), for a cumulative occupancy value of x, $\beta_3 + \beta_7 x$ represents the immediate effect of the SMOCC initiation, $\beta_4 + \beta_8 x$ represents the sustained linear effect of the SMOCC initiation, and $\beta_5 + \beta_9 x$ represents the sustained quadratic effect of the SMOCC initiation. Additionally, β_6 $(\beta_6 + \beta_7 + \beta_8 p_t + \beta_9 p_t^2)$ represents the effect of

© 2025 Richert ME et al. *JAMA Network Open.*

cumulative occupancy before (p_t weeks after) the SMOCC initiation. Interpretations under models (1)-(3) can be made in a similar fashion.

The maximum likelihood method was used to estimate the unknown parameters and the Newey-West method[4] was used to estimate the standard errors to account for potential autocorrelation within time series data. The model with the smallest Akaike Information Criterion was selected.[5]

We conducted a residual model diagnosis to assess the linearity of occupancy stress. The plot of deviance residuals against stress (eFigure 4) shows that the residuals are evenly distributed around zero, with no obvious pattern, indicating the linearity assumption is reasonable.

We conducted the ITS analyses for the entire cohort and subgroups defined by variables such as urbancity, age, transport mode, level of care, and initial patient acuity. All analyses were conducted using the R software, version 4.4.2 (R Core Team, 2023).

eTable 5: Overall Interhospital Transfer Characteristics During Study Period

		Total Overall Transfers No (%)	Final Cohort Transfer from Eight Study States No (%)		Excluded Transfers No (%)
			Pre-SMOCC	Post-SMOCC	
**Encounters (n)**		4,549,789	120,631	321,078	4,108,080
**Age (years/old)**	**Median [IQR]**	62.0 [45.0, 74.0]	61.0 [43.0, 73.0]	61.0 [44.0, 74.0]	62.0 [45.0, 74.0]
**Age Category (years/old)**	**18-44**	1,128,930 (24.8%)	32,102 (26.6%)	81,987 (25.5%)	1,014,841 (24.7%)
	**45-61**	1,130,185 (24.8%)	30,323 (25.1%)	79,561 (24.8%)	1,020,301 (24.8%)
	**62-73**	1,126,917 (24.8%)	29,599 (24.5%)	78,799 (24.5%)	1,018,519 (24.8%)
	**74+**	1,163,757 (25.6%)	28,607 (23.7%)	80,731 (25.1%)	1,054,419 (25.7%)
**Gender**	**Female**	2,155,825 (47.4%)	56,494 (46.8%)	152,859 (47.6%)	1,946,472 (47.4%)
	**Male**	2,350,885 (51.7%)	62,678 (52.0%)	165,304 (51.5%)	2,122,903 (51.7%)
	**Unreported**	43,079 (0.9%)	1,459 (1.2%)	2,915 (0.9%)	38,705 (0.9%)
**Urbanicity**	**Rural**	1,040,804 (22.9%)	30,056 (24.9%)	84,251 (26.2%)	926,497 (22.6%)
	**Urban**	3,008,961 (66.1%)	88,938 (73.7%)	231,019 (72.0%)	2,689,004 (65.5%)
	**Unreported**	500,024 (11.0%)	1,637 (1.4%)	5,808 (1.8%)	492,579 (12.0%)

© 2025 Richert ME et al. *JAMA Network Open.*

Region	Northeast	454,983 (10.0%)	0 (0.0%)	0 (0.0%)	454,983 (11.1%)
	South	1,756,703 (38.6%)	39,922 (33.1%)	150,150 (46.8%)	1,566,631 (38.1%)
	Midwest	847,771 (18.6%)	0 (0.0%)	0 (0.0%)	847,771 (20.6%)
	West	1,039,073 (22.8%)	80,709 (66.9%)	170,928 (53.2%)	787,436 (19.2%)
	Unreported	451,259 (9.9%)	0 (0.0%)	0 (0.0%)	451,259 (11.0%)
Initial Acuity	Lower Acuity (Green)	2,336,733 (51.4%)	49,160 (40.8%)	146,126 (45.5%)	2,141,447 (52.1%)
	Emergent (Yellow)	822,110 (18.1%)	20,536 (17.0%)	52,553 (16.4%)	749,021 (18.2%)
	Critical (Red)	203,958 (4.5%)	7,175 (5.9%)	21,217 (6.6%)	175,566 (4.3%)
	Dead (Black)	1,751 (0.0%)	25 (0.0%)	73 (0.0%)	1,653 (0.0%)
	Unreported	1,185,237 (26.1%)	43,735 (36.3%)	101,109 (31.5%)	1,040,393 (25.3%)
Final Acuity	Lower Acuity (Green)	2,345,619 (51.6%)	54,572 (45.2%)	147,595 (46.0%)	2,143,452 (52.2%)
	Emergent (Yellow)	694,725 (15.3%)	19,226 (15.9%)	41,249 (12.8%)	634,250 (15.4%)
	Critical (Red)	147,975 (3.3%)	6,068 (5.0%)	15,077 (4.7%)	126,830 (3.1%)
	Dead (Black)	1,761 (0.0%)	30 (0.0%)	96 (0.0%)	1,635 (0.0%)
	Unreported	1,359,709 (29.9%)	40,735 (33.8%)	117,061 (36.5%)	1,201,913 (29.3%)
Transport Mode	Air	282,640 (6.2%)	10,407 (8.6%)	35,306 (11.0%)	236,927 (5.8%)
	Ground	4,267,149 (93.8%)	110,224 (91.4%)	285,772 (89.0%)	3,871,153 (94.2%)
Level Of Care	Advanced Life Support	2,819,056 (62.0%)	86,549 (71.7%)	192,003 (59.8%)	2,540,504 (61.8%)
	Basic Life Support	1,036,955 (22.8%)	11,991 (9.9%)	45,917 (14.3%)	979,047 (23.8%)
	Specialty Critical Care	693,778 (15.2%)	22,091 (18.3%)	83,158 (25.9%)	588,529 (14.3%)
Season	Fall	1,169,195 (25.7%)	27,373 (22.7%)	100,645 (31.3%)	1,041,177 (25.3%)
	Spring	814,802 (17.9%)	13,297 (11.0%)	63,964 (19.9%)	737,541 (18.0%)
	Summer	1,245,796 (27.4%)	66,235 (54.9%)	76,488 (23.8%)	1,103,073 (26.9%)
	Winter	868,737 (19.1%)	13,726 (11.4%)	79,981 (24.9%)	775,030 (18.9%)

© 2025 Richert ME et al. *JAMA Network Open.*

	**Unreported**	451,259 (9.9%)	0 (0.0%)	0 (0.0%)	451,259 (11.0%)
**Transport Time**	**Median (min) [IQR]**	56.0 [39.0, 81.0]	54.9 [38.7, 82.8]	55.0 [39.2, 82.5]	56.0 [39.0, 81.0]
	**Unreported**	20,058 (0.4%)	428 (0.4%)	1,136 (0.4%)	18,494 (0.5%)

eTable 6: Full Interrupted Times Series Analysis of Aggregated Eight SMOCC Study States

	**Rate Ratio (95% CI)**	**p-value**
(Intercept)	3523.59 (2571.76- 4827.70)	<0.0001
Effect of Time (by week)	1.0272 (1.0105 - 1.0442)	0.0013
Quadratic Effect of Time (by week)	1.0004 (1.0001 - 1.0007)	0.0091
Immediate Post SMOCC Effect (D)	1.3516 (1.0511 - 1.7381)	0.0189
Long-term Post-SMOCC Effect (P)	0.9364 (0.9035 - 0.9705)	0.0003
Hospital Occupancy Stress Before SMOCC	0.9887 (0.9377 - 1.0426)	0.6757
Spring	1.5964 (1.1800 - 2.1598)	0.0024
Summer	1.4417 (1.0699 - 1.9426)	0.0162
Fall	1.0493 (0.6906 - 1.5944)	0.8216
Interaction of Immediate Post SMOCC Effect (D) and Hospital Occupancy Stress	1.0695 (0.7008 - 1.6322)	0.7553
Interaction of Long-term Post SMOCC Effect (P) and Hospital Occupancy Stress	1.0037 (0.9959 - 1.0116)	0.3548

eTable 7: Hospital Occupancy Stress Effect on Interhospital Transfer (IHT) Rate Over Time

	**Rate Ratio (95% CI)**	**p-value**
Hospital Occupancy Stress Before SMOCC	0.99 (0.94 - 1.04)	0.68
Hospital Occupancy Stress Immediately After SMOCC	1.06 (0.70 - 1.61)	0.79
**Hospital Occupancy Stress at 40 Weeks Post SMOCC**	**1.23 (1.06 - 1.42)**	**0.007**

© 2025 Richert ME et al. *JAMA Network Open.*

eTable 8: Sensitivity Analysis with Unweighted Hospital Occupancy Stress Effect on Interhospital Transfer (IHT) Rate Over Time

	Rate Ratio (95% CI)	p-value
Hospital Occupancy Stress Before SMOCC	0.9727 (0.9286 - 1.0189)	0.2429
Hospital Occupancy Stress Immediately After SMOCC	1.1901 (0.9310 - 1.5213)	0.1648
**Hospital Occupancy Stress at 20 Weeks Post SMOCC**	**1.1800 (1.0098 - 1.3789)**	**0.0373**
**Hospital Occupancy Stress at 40 Weeks Post SMOCC**	**1.1699 (1.0655 - 1.2847)**	**0.0010**

eTable 9: Sensitivity Analysis with Unweighted Hospital Occupancy Stress for Full Interrupted Times Series Analysis of Aggregated Eight SMOCC Study States

	Rate Ratio (95% CI)	p-value
(Intercept)	3479.8 (2527.6 - 4790.8)	<0.0001
Effect of Time (by week)	1.0307 (1.0151 - 1.0466)	0.0001
Quadratic Effect of Time (by week)	1.0004 (1.0002 - 1.0007)	0.0012
Immediate Post SMOCC Effect (D)	1.4773 (1.1689 - 1.8672)	0.0011
Long-term Post-SMOCC Effect (P)	0.9311 (0.8997 - 0.9635)	0.0000
Hospital Occupancy Stress	0.9727 (0.9286 - 1.0189)	0.2429
Spring	1.6307 (1.2409 - 2.1429)	0.0005
Summer	1.5665 (1.1551 - 2.1244)	0.0039
Fall	0.9757 (0.6493 - 1.4660)	0.9056
Interaction of Immediate Post SMOCC Effect (D) and Hospital Occupancy Stress	1.2235 (0.9450 - 1.5840)	0.1258
Interaction of Long-term Post SMOCC Effect (P) and Hospital Occupancy Stress	0.9996 (0.9945 - 1.0046)	0.8687

© 2025 Richert ME et al. *JAMA Network Open.*

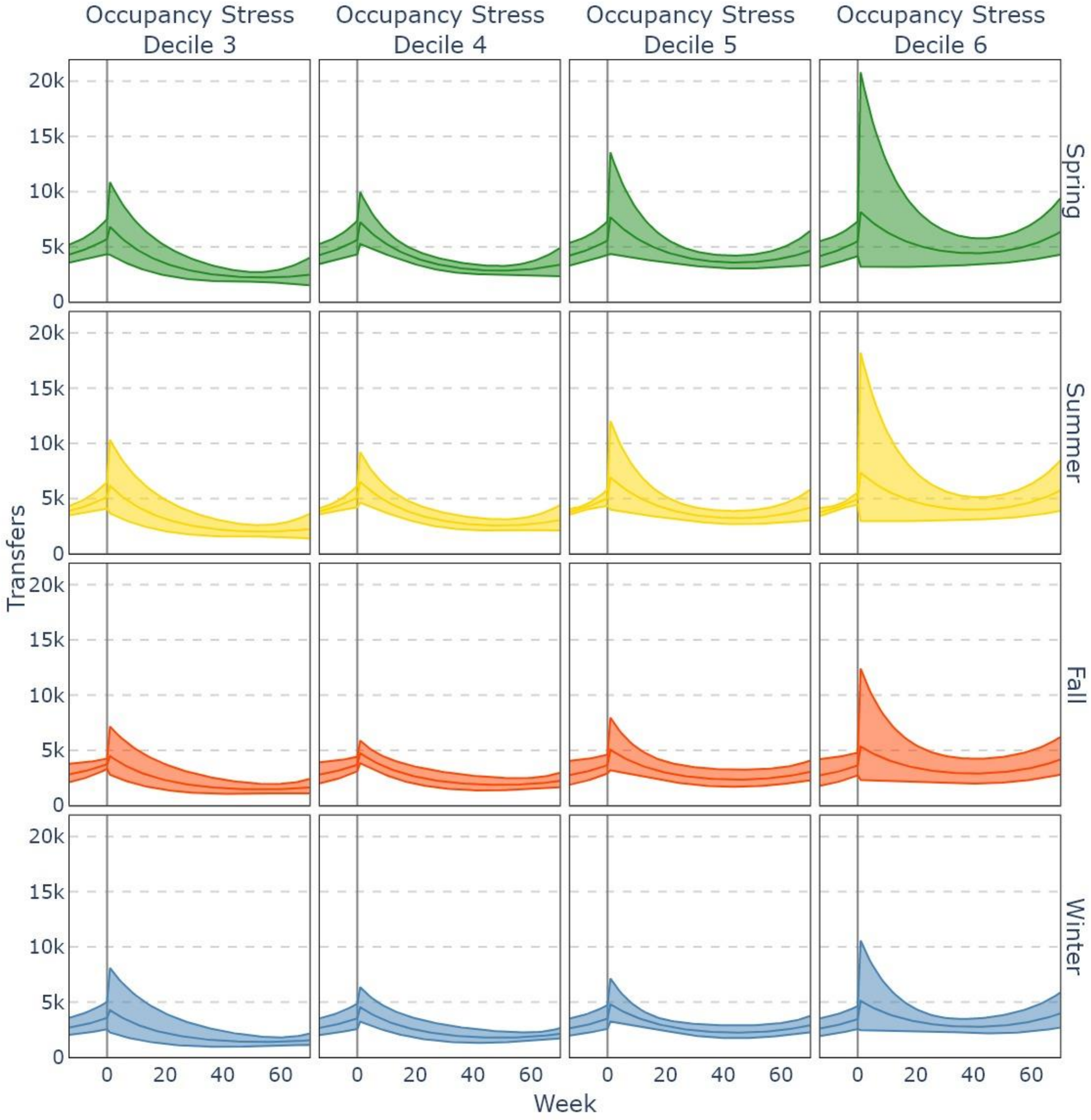

eFigure 2: Cumulative Interhospital Transfers and 95% pointwise confidence bands over time since SMOCC initiation by Hospital Occupancy Stress decile level and by season.

© 2025 Richert ME et al. *JAMA Network Open.*

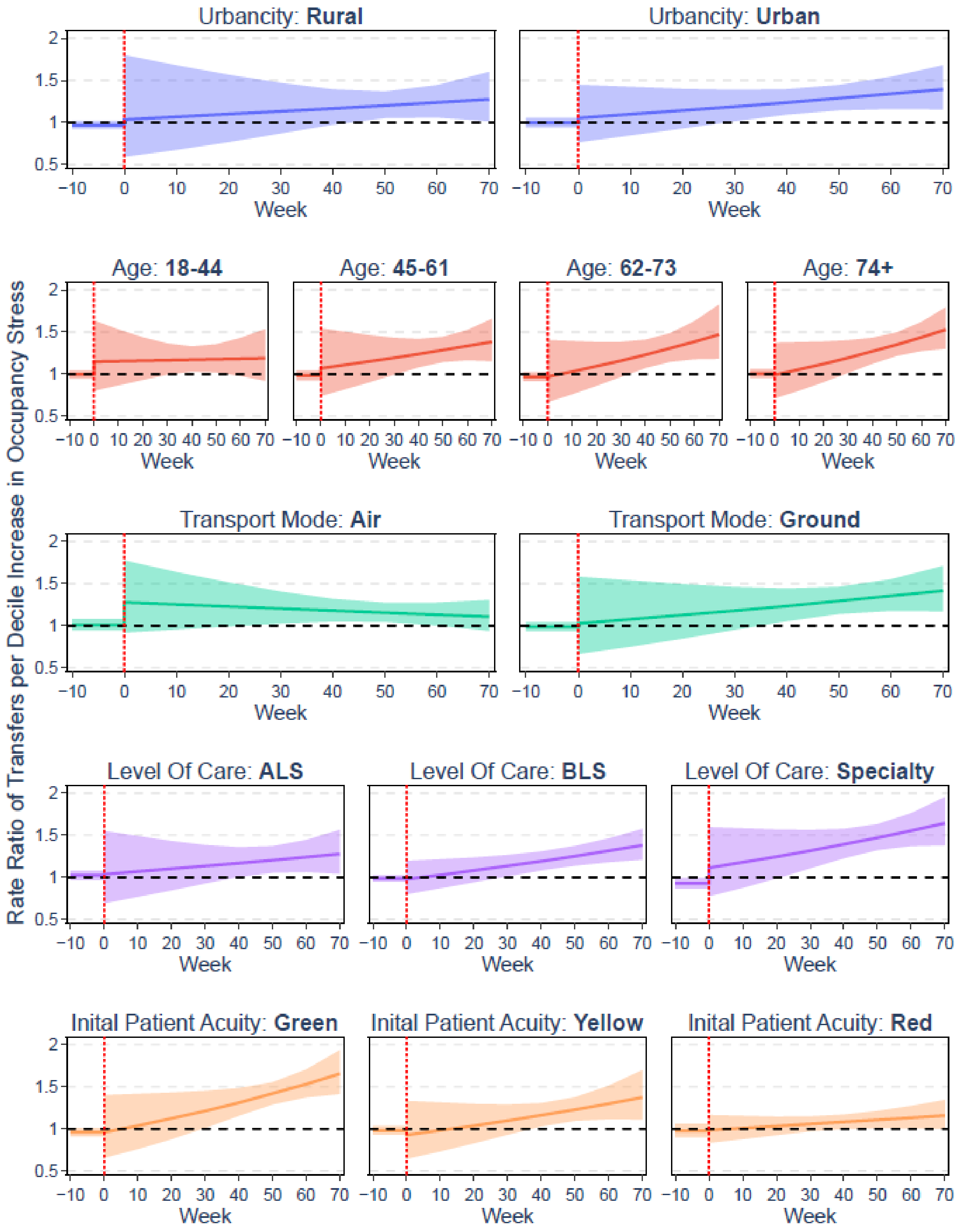

eFigure 3: Subgroup Analysis for Change in Interhospital Transfers with Increasing Occupancy Stress and Interaction by Time Since SMOCC Establishment. Demonstrating change in interhospital transfer rates (denoted by rate ratio) associated with one unit increase in stress occupancy decile.

© 2025 Richert ME et al. *JAMA Network Open.*

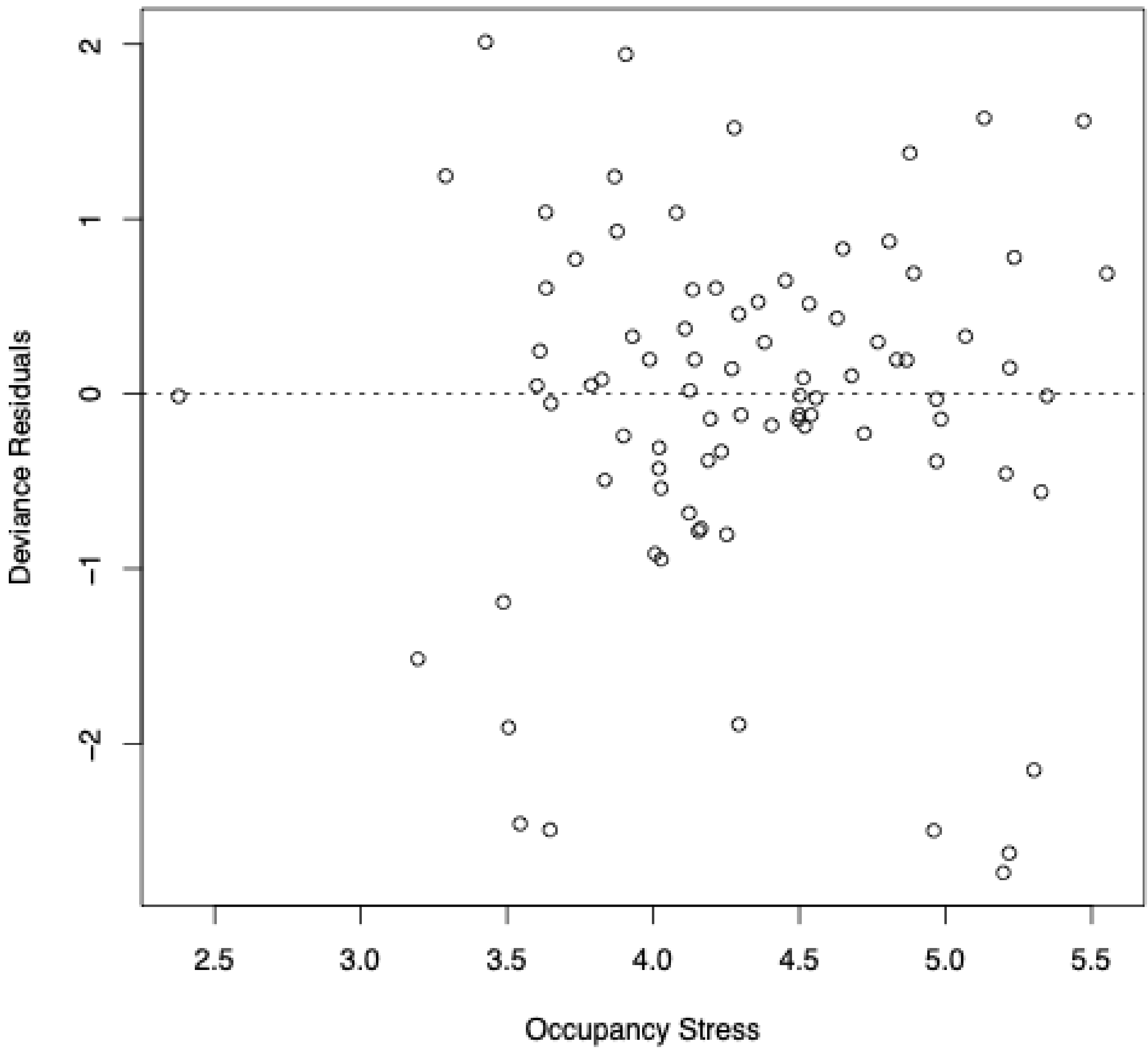

eFigure 4. Plot of deviance residuals against occupancy stress. The random scatter of points around zero suggests that the relationship between occupancy stress and IHT is adequately captured by the fitted negative binomial regression model.

© 2025 Richert ME et al. *JAMA Network Open.*

References

1. NEMSIS Data Dictionary v 3.4.0. Accessed August 15, 2025. https://nemsis.org/nemsis-data-dictionary-v3-4-0/
2. U.S. Department of Health and Human Service. COVID-19 Reported Patient Impact and Hospital Capacity by Facility. Published online 2024. https://healthdata.gov/Hospital/COVID-19-Reported-Patient-Impact-and-Hospital-Capa/anag-cw7u
3. French G. Impact of Hospital Strain on Excess Deaths During the COVID-19 Pandemic — United States, July 2020–July 2021. *MMWR Morb Mortal Wkly Rep*. 2021;70. doi:10.15585/mmwr.mm7046a5
4. Newey WK, West KD. A Simple, Positive Semi-Definite, Heteroskedasticity and Autocorrelationconsistent Covariance Matrix. Published online April 1, 1986. Accessed March 26, 2025. https://papers.ssrn.com/abstract=225071
5. Akaike H. A new look at the statistical model identification. *IEEE Trans Automat Contr*. 1974;19(6):716-723. doi:10.1109/TAC.1974.1100705

© 2025 Richert ME et al. *JAMA Network Open.*
