# Supplementary material for: Statewide Transfer Coordination and Patient Transfer Rates Among Hospitals During Occupancy Stress
Source: JAMA Netw Open. 2025 Dec 1;8(12):e2546002. doi: 10.1001/jamanetworkopen.2025.46002 (PMC12670198; doi:10.1001/jamanetworkopen.2025.46002)
Supplement: Supplement 2. — Data Sharing Statement [file jamanetwopen-e2546002-s002.pdf]

Data Sharing Statement

Richert. Statewide Transfer Coordination and Patient Transfer Rates Among Hospitals During Occupancy Stress. *JAMA Netw Open*. Published December 01, 2025.
doi:10.1001/jamanetworkopen.2025.46002

Data
Data available: Yes
Data types: Deidentified participant data
How to access data: NEMSIS data can be requested using the following link: Request Research Data - NEMSIS
When available: With publication

Supporting Documents
Document types: None

Additional Information
Who can access the data: Anyone requesting the data
Types of analyses: For any purpose
Mechanisms of data availability: NEMSIS data can be requested using the following link: Request Research Data - NEMSIS
